# Oxidation Mechanism in Bigels and Emulgels—Challenges and Solutions

**DOI:** 10.3390/molecules31060970

**Published:** 2026-03-13

**Authors:** Szymon Juchniewicz, Joanna Harasym

**Affiliations:** 1 Adaptive Food Systems Accelerator-Science Centre, Wroclaw University of Economics and Business, Komandorska 118/120, 53-345 Wroclaw, Poland; 2 Department of Biotechnology and Food Analysis, Wroclaw University of Economics and Business, Komandorska 118/120, 53-345 Wroclaw, Poland

**Keywords:** bigels, emulgels, interfacial oxidation, antioxidant strategies, association colloids, pickering emulsions

## Abstract

Nutritionally crucial unsaturated fatty acids, especially rich in high omega-3 bonds, are very prone to oxidation. This phenomenon makes oxidation stability a substantial challenge in every formulation, especially those which contain or at some stage of preparation contain water. Bigels and emulgels, which represent promising structured lipid systems for replacing saturated and trans fats in food formulations, pose significant oxidative stability challenges. This review examines oxidation mechanisms in such biphasic systems. Oxidation in bigels and emulgels proceeds through both free-radical-mediated autoxidation and metal-ion-catalysed pathways, with the oil–water interface serving as the primary reaction zone where pro-oxidants concentrate, and lipid substrates become accessible. Structural configuration critically determines oxidative stability, following the sequence W/O bigel > bicontinuous bigel > O/W bigel. The high viscosity of gel matrices provides substantial protection by restricting radical mobility and oxygen diffusion. Mass transfer occurs via diffusion, collision–exchange–separation, and micelle-assisted mechanisms, with association colloids forming localized interfaces that accelerate oxidation. Thermal processing presents particular challenges, as temperatures above 50 °C disrupt most gel structures and accelerate oxidative degradation. Effective protective strategies include interfacial engineering with emulsifiers to reduce oil–water interfacial tension, incorporation of natural antioxidants (e.g., phenolic compounds and tocopherols), and synergistic antioxidant combinations. This review provides a mechanistic framework for formulating oxidatively stable bigels and emulgels suitable for food applications.

## 1. Introduction

**Figure 1:**
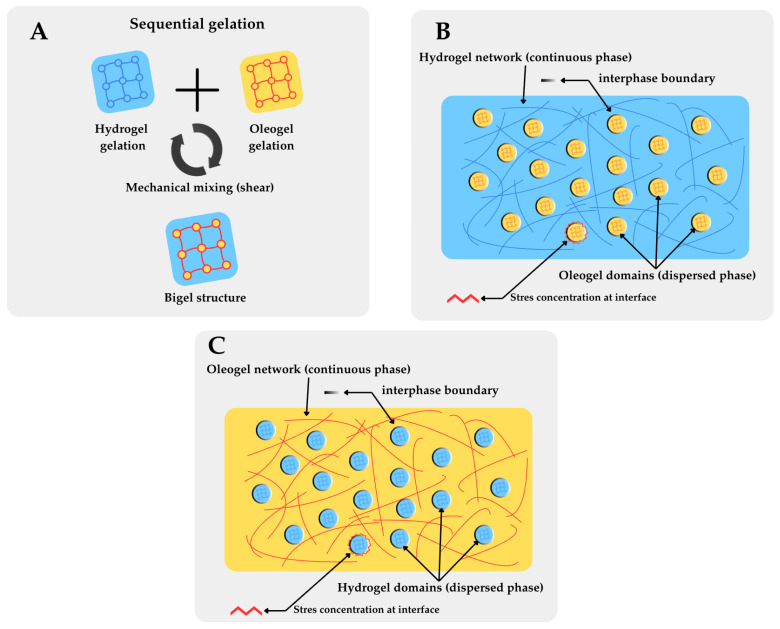
Bigel systems (**A**) independent gelation of each phase followed by mechanical mixing (**B**) oleogel-in-hydrogel or (**C**) hydrogel-in-oleogel.

Bigels and emulgels are structured lipid systems designed and validated as viable alternatives to traditional saturated and trans fats in food formulations [1,2]. They are specifically designed to overcome the limitations of conventional fat replacers, which often fail to replicate the complex sensory and rheological properties of fats in food matrices. Bigels are mixtures of two gel systems (Figure 1), a hydrogel phase (aqueous) and an oleogel phase (lipid), whose proportions when changed allow for the achievement of tailored physical and rheological properties [3,4]. Mixing two, already set, gel systems results in synergistic interaction between the hydrogel and oleogel phases, enabling the achievement of notable structural stability and texture profile compared to homogenous gel systems.

The oleogel phase typically consists of vegetable oils structured using oleogelators such as waxes (beeswax, candelilla wax, rice bran wax), monoglycerides, or ethylcellulose, while the hydrogel phase incorporates polysaccharides (starch, alginate, carrageenan) or proteins (gelatin, whey protein) as gelling agents [5,6]. Emulgels, conversely, represent an emulsion matrix, which is further stabilised by hydrocolloids, while oil droplets are dispersed within a continuous gel matrix. These structured lipids offer significant advantages, such as reduced caloric content through partial fat replacement in products; improved oxidative stability using structured matrices; enhanced nutritional profiles through the incorporation of bioactive compounds; and appealing texture and mouthfeel characteristics that closely approximate those of traditional fats [7,8].

Although bigels and emulgels are both considered semi-solid biphasic systems of oil and aqueous components, their matrix organisation differs fundamentally because of their different mechanisms of formation. The resulting functional properties, especially oxidative stability (Figure 1), are distinct. Bigels are composed of two independently pre-formed gel phases—a hydrogel and an oleogel—subsequently combined through mechanical mixing (shearing) to produce a composite structure in which discrete gel domains coexist, separated by an interphase boundary [3,4,9]. Depending on the oleogel-to-hydrogel volume ratio (φ), bigels adopt one of three morphologies—oleogel-in-hydrogel (O/W, when φ < 1), hydrogel-in-oleogel (W/O, when φ > 1), or bicontinuous (when φ ≈ 1)—in which overlapping continuous networks of both phases are present [10,11,12].

Emulgels, in contrast, are formed from a single emulsification step: oil droplets are first dispersed in an aqueous phase (or vice versa) using emulsifiers or surface-active agents, and the resulting emulsion is then immobilised within a gel network formed by crosslinking biopolymers such as proteins (whey protein, gelatin, casein, soy protein), polysaccharides (xanthan gum, starch, alginate, pectin), or their combinations [8,13,14]. The resulting structure consists of individual oil droplets trapped within a continuous gelled matrix, rather than macroscopic gel domains.

These structural and formation differences carry important consequences for oxidative stability that are central to this review. In bigels, the interphase boundary between macroscopic gel domains has a relatively lower total surface area compared to finely dispersed emulgel droplets, but the interphase region remains the primary site where pro-oxidant species concentrate, and lipid substrates become accessible [10,15]. The tuneable oleogel-to-hydrogel ratio in bigels allows direct manipulation of the continuous phase identity (oil-continuous W/O vs. water-continuous O/W), which determines the oxidation stability sequence discussed in Section 2.5 [10].

In emulgels, the finer droplet dispersion results in a larger total oil–water interfacial area, which generally increases oxidation susceptibility [13,15]; however, the gel network surrounding each droplet provides a physical diffusion barrier that restricts the movement of pro-oxidants from the aqueous phase toward unsaturated lipids, partially counteracting the larger interfacial exposure [13,14]. Furthermore, the interfacial film composition in emulgels—governed by the emulsifier or biopolymer used during the emulsification step—directly determines the accessibility of metal ions and oxygen to the lipid phase, providing an additional control parameter for oxidative stability that is less readily manipulated in bigels where the interface forms through mechanical disruption rather than controlled adsorption [11,15,16].

Throughout this review, “bigel” refers exclusively to systems produced by mixing two pre-formed gels, while “emulgel” (also termed “emulsion gel” in some literature) refers to systems in which emulsion droplets are immobilised within a gelled continuous phase. Where studies report on both system types or on systems with intermediate characteristics, this is noted explicitly.

Fat mimicry in bigels and emulgels results from two main pathways. First, the oleogel phase provides the lubricating and textural properties essential for fat functionality, while the hydrogel phase contributes to structural integrity and moisture retention [3]. Second, the hydrocolloid network surrounding oil droplets in emulgels creates a complex diffusion path that limits moisture loss and oxidative degradation, thereby extending shelf-life and maintaining product quality. The dual-phase structure distinguishes bigels and emulgels from conventional fat replacers, which typically employ either cellulose-based or protein-based systems alone [17]. Both systems offer the potential to create trans-fat-free products with elevated levels of unsaturated fatty acids (UFA), while facing the typical oxidation issues associated with UFA.

Understanding the oxidation of biphasic emulsions with varying physical states is critical. Semi-solid emulsions with high oil content, such as mayonnaise and margarine, are prone to lipid oxidation. Unfortunately, fewer studies have focused on lipid oxidation in these gelled emulsions than in fluid emulsions [15]. Some research indicates that gelled emulsions may inhibit lipid oxidation compared with fluid emulsions because their high viscosity can lower the diffusion rates of oxygen and pro-oxidants [13].

This review aims to provide a comprehensive mechanistic understanding of oxidation phenomena in bigel and emulgel systems, addressing a critical gap in the literature: oxidation studies have predominantly focused on fluid emulsions rather than gelled biphasic structures. By synthesising current knowledge on mass transfer mechanisms, interfacial chemistry, and the structural dependence of oxidative stability (W/O > bicontinuous > O/W), this review also critically evaluates both synergistic and antagonistic antioxidant combinations specific to bigel matrices.

Existing reviews have thoroughly addressed lipid oxidation in fluid emulsions [13,16,18,19] structural and rheological properties of oleogels or hydrogels independently [7,9,20,21], or the application potential of bigels as fat replacers with oxidative stability as one parameter among many [4,6,9]. However, none has provided an integrated mechanistic framework connecting interfacial oxidation chemistry, mass transfer constraints, and structure-dependent stability hierarchies specific to gelled biphasic systems where the interphase boundary—not merely the oil composition—governs oxidative outcomes.

The field of structured lipid systems has advanced rapidly in recent years, with several developments directly relevant to the oxidative stability challenges addressed in this review. Recent studies have introduced molecular dynamics simulations to elucidate bigel component interactions at the molecular level [9], and MRI-based techniques to visualise water mobility and phase integrity in beeswax–gelatin bigel systems before and after freeze–thaw stress [12].

Cellulose nanofiber-reinforced bigels have demonstrated enhanced thermal stability and textural properties [22], while algal oil-based bigel co-delivery systems have revealed structure-dependent antioxidant synergies, establishing the oxidative stability hierarchy W/O > bicontinuous > O/W [10]. The emulsion-template approach to oleogelation has emerged as a promising low-temperature alternative to conventional direct structuring, reducing processing-induced oxidation [23,24]. Comprehensive reviews have also consolidated the potential of bigels as solid fat replacers for food applications, though with limited attention to oxidation mechanisms [9]. These advances provide the context for the present mechanistic synthesis.

This is a narrative review, based on the databases consulted: Web of Science, Scopus, PubMed, and Google Scholar within the primary time frame: 2014–2026, with emphasis on publications from 2021–2026. Search terms: combinations of “bigel,” “emulgel,” “oleogel,” “lipid oxidation,” “oxidative stability,” “structured lipids,” “fat replacer,” “interfacial oxidation,” “antioxidant,” “mass transfer,” and “emulsion gel.” The chosen inclusion criteria were peer-reviewed English-language articles reporting on oxidation mechanisms, oxidative stability assessment, or antioxidant strategies in structured lipid systems, with priority given to studies involving biphasic gel systems relevant to food applications. Articles focusing exclusively on pharmaceutical or cosmetic applications without food-relevant oxidation data were excluded.

## 2. Oxidation Phenomena and Oxidative Stability Challenges

### 2.1. Lipid Oxidation Mechanisms in Structured Lipids

**Figure 2:**
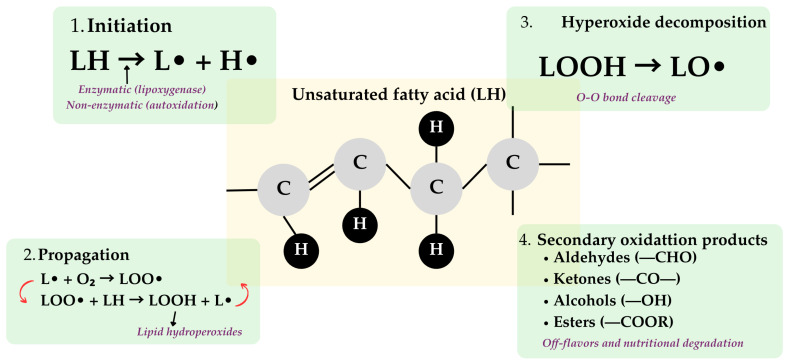
Unsaturated fatty acids oxidation pathways.

Oxidation represents the most significant challenge in developing shelf-stable bigels and emulgels, as the incorporation of liquid oil into gel matrices increases the surface area available for oxidative attack [1,13,16]. The oxidation of unsaturated fatty acids occurs through both enzymatic (lipoxygenase-catalyzed) and non-enzymatic (free radical-mediated autoxidation) pathways (Figure 2).

Any structured matrix creates obstacles to the transport of molecules. Specifically in structured lipids, the gel matrix can either protect the oil through diffusion limitation or, conversely, concentrate pro-oxidant species at the oil–water interface, accelerating degradation [2,18,25].

The oxidation process begins with the abstraction of hydrogen atoms from unsaturated fatty acids, forming lipid radicals (L•) that react with molecular oxygen to generate peroxyl radicals (LOO•). These peroxyl radicals propagate the oxidation chain by abstracting hydrogen from adjacent lipid molecules, creating a self-sustaining cascade that produces primary oxidation products (hydroperoxides, and lipid hydroperoxides-LOOH) and secondary products (aldehydes, ketones, alcohols) responsible for off-flavors and nutritional degradation [16,19,26]. The primary pathway of lipid hydroperoxide degradation involves the cleavage of the weak O–O bond in the LOOH structure to form an alkoxyl radical (LO•). Scission reaction of this LO• radical results in the formation of a variety of volatile compounds, including aldehydes, ketones, alcohols, and esters that contribute to the characteristic off-flavors of lipid oxidation (Figure 2).

Chen et al. (2022) [15] demonstrated that higher susceptibility of lipids to oxidation (>2.5 times) was observed in biphasic O/W and W/O emulgels than in soybean oil suggesting either processing or interfacial region contribution [10]. Additionally, in the heterogeneous emulsion systems, W/O emulgels exhibited greater oxidation resistance than O/W emulgels [15,18].

Wei et al. demonstrated that in oleogel-in-hydrogel (O/W) bigels without added antioxidants, peroxide values (PV) reached maximum levels of 107.2 mmol/kg oil and thiobarbituric acid reactive substances (TBARS) values of 160.8 μmol/kg oil after 35 days of storage at 25 °C, with oxidation products accumulating most rapidly due to the smaller droplet size and larger interfacial area exposure [10].

### 2.2. Mass Transfer of Oxidation Products in Structured Systems

**Figure 3:**
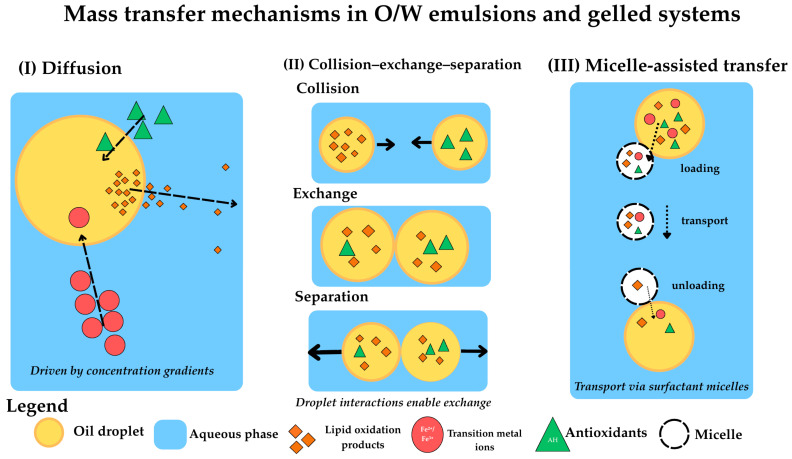
O/W emulsions mass transfer phenomena.

The transport of lipid oxidation products, transition metal ions, and antioxidants is a crucial process that is often overlooked in the determination of oxidation rates in emulsions, particularly in gelled systems [13,19,27]. There are three mechanisms of mass transfer in O/W emulsions, which are involved: (I) diffusion, (II) collision–exchange–separation, and (III) micelle-assisted transfer (Figure 3).

In the diffusion mechanism, molecules diffuse from one oil droplet to another through the water phase, while in the collision–exchange–separation mechanism, when oil droplets collide, they exchange material. The micelle-assisted transfer mechanism includes molecules that are solubilised in micelles within the water phase and then transferred between lipid droplets [13,28,29].

Considering diffusion properties, three types of compounds can be produced during lipid oxidation: (I) water-soluble compounds, such as carbonyl compounds; (II) surface-active compounds (e.g., LOOHs); and (III) hydrophobic compounds (e.g., lipoperoxy radicals).

Critically, peroxyl radicals induced by 2,2′-azobis(2,4-dimethylvaleronitrile) (AMVN) could transfer between oil droplets, while no transfer was observed for alkoxyl radicals produced by di-tertbutyl peroxide [13,16]. This is due to the shorter lifetimes of alkoxy radicals (10^−6^ s) compared to peroxyl radicals (0.5–7 s), which limit their transfer between oil droplets [13].

**Figure 4:**
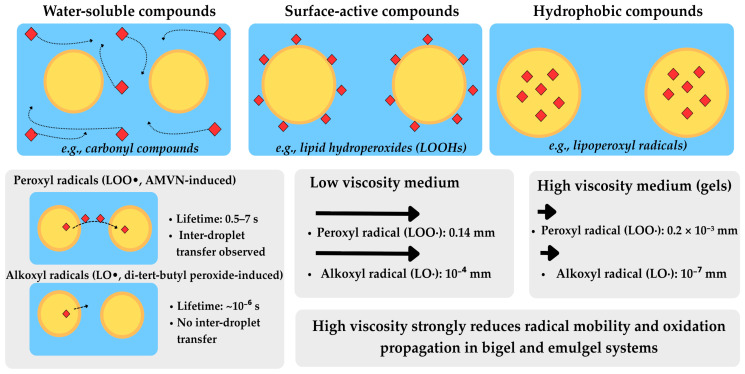
Propagation of the compounds resulting from lipid oxidation in the mixed matrices.

Importantly, the distance travelled by radicals has crucial implications for oxidation in solidified gel systems. A peroxyl radical can cross much longer distances (0.14 mm in a non-viscous medium and 0.2 × 10^−3^ mm in a viscous one) than alkoxyl radicals (10^−4^ mm in a non-viscous medium and 10^−7^ mm in a viscous one). This explains why the high viscosity of bigel and emulgel systems provides substantial protection against oxidation—by strongly reducing radical mobility and the propagation of oxidation reactions [13,15,30]. Propagation of the compounds resulting from lipid oxidation in the mixed matrices is explained in Figure 4.

### 2.3. Pro-Oxidant Factors and Interfacial Mechanisms

Bigel and emulgel structures are subject to multiple factors and conditions that accelerate the oxidation of incorporated fat [13,16,31]. Transition metal ions, particularly iron and copper, are among the main pro-oxidant factors in foods. Metal ions can contribute to the initiation reaction and are key catalysts for the degradation of lipid hydroperoxides, leading to the formation of LOO• or LO• radicals through the reaction of Mn^+1^ (e.g., ferric Fe^III^ and cupric Cu^II^) or Mn (e.g., ferrous Fe^II^ and cuprous Cu^I^) transition metal ions, respectively, with hydroperoxides [16,32,33].

**Figure 5:**
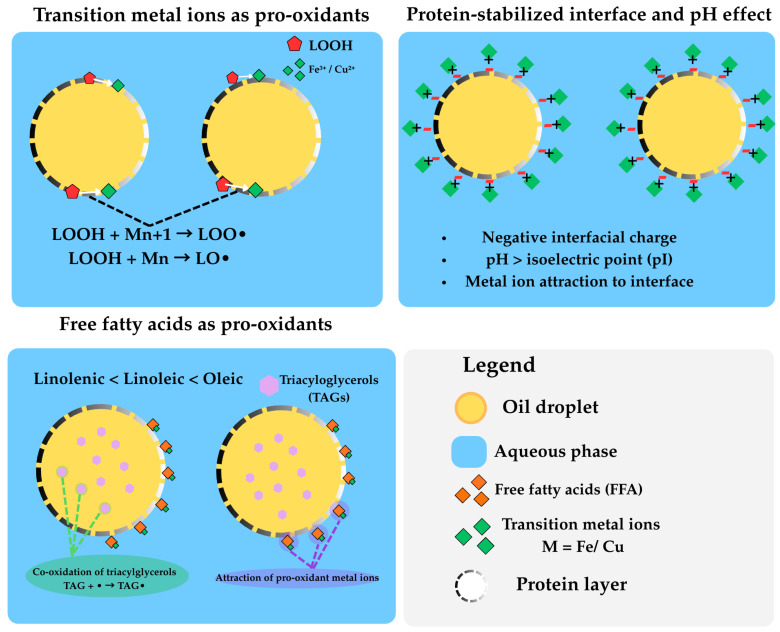
Oxidation of lipids at the interphase.

The negative charge at the interface enhances oxidation by attracting metal ions toward the oil phase. Additionally, the pH of the emulsion alters the charge of the proteins. When the pH values are higher than the isoelectric point of proteins, the charge of the interfacial region stabilised by proteins is negative. This results in proteins enhancing the oxidation rate of emulsions at high pH values due to charge dynamics at the interface [13,34,35]. Also, free fatty acids that remain in the oil phase after refining, affect lipid oxidation rates, with pro-oxidant effects in the order linolenic < linoleic < oleic. This pro-oxidant effect is related to the ability of free fatty acids to attract pro-oxidant metal ions and to co-oxidize triacylglycerols in bulk oil (Figure 5).

### 2.4. Physicochemical Mechanisms of Oxidation at the Interphase

The interphase region in bigels and emulgels is a critical zone where oxidation processes accelerate compared to the bulk phases [13,25,32]. The interface between the hydrophilic hydrogel phase and the hydrophobic oleogel phase forms an electrochemical environment with pro- and antioxidant species converging. In oil-in-water emulsions, oxidative stability is strongly influenced by the physicochemical properties of the surfactant layer at the oil droplet interface, as chelating agents and salts can interfere with the transport of oxidation products, thereby altering lipid oxidation rates [32,36]. The electrical potential (zeta potential) and the relaxation time of surfactant-coated lipid droplets are therefore critical parameters affecting how metal ions and other pro-oxidant species access the oil phase. When chelating agents are incorporated, they effectively reduce the adsorption of metal ions to the droplet surfaces, thereby preventing catalytic oxidation [31,32]. In contrast, monovalent salts such as sodium chloride or potassium chloride slightly increase lipid oxidation rates by altering the physical properties of the surfactant layer, as ionic strength and electrostatic interactions fundamentally impacts oxidation kinetics at the interphase.

Both autocatalytic and metal ion-catalyzed pathways are observed at interfacial regions [16,18]. Even trace concentrations of endogenous iron and copper ions (at levels as low as 1.99 and 0.86 ppm, respectively) present in aqueous phases are sufficient to dramatically accelerate lipid peroxidation [32]. These transition metal ions participate in Fenton-like reactions that generate highly reactive hydroxyl radicals and other reactive oxygen species (ROS), which then initiate lipid radical chain reactions at the interface [31,32,33]. The spatial arrangement of the surfactant molecules at the oil droplet surfaces becomes crucial because it determines the accessibility of these pro-oxidant species to fatty acid substrates within the oil phase. Additionally, the location of antioxidants relative to the interphase affects their protective effects—polyphenolic compounds and other natural antioxidants must be positioned to intercept peroxyl radicals before they can propagate the chain reaction, or alternatively, they must reduce the accessibility of catalytic metal ions to the lipid phase [25,37,38]. The clue is that the lack of metal ions is practically impossible to avoid in food formulations, especially since those metals are also nutritionally important. Therefore, metal-ion-based oxidation of lipids is a basis in foods.

### 2.5. Structural Dependence and Oxidation Stability Sequence

Seems that in bigels and emulgels, structural configuration is a main factor impacting oxidative stability [10,11,15]. Studies comparing different bigel architectures revealed that oxidation stability follows the sequence: W/O bigel > bicontinuous bigel > O/W bigel [10]. In W/O systems containing algal oil, the fat crystal network formed a higher-viscosity and denser interface layer that hindered oxygen penetration and diffusion. After 35 days of storage, W/O bigels exhibited the lowest PV (14.2 mmol/kg oil) and TBARS (18.6 μmol/kg oil) values when co-encapsulating astaxanthin and ascorbic acid, representing approximately 87% and 88% reductions compared to unprotected O/W systems, respectively [10]. The protective effect of gel network density has been demonstrated in single-gelator systems, where agarose-structured oleogels showed 34% less aroma decay than pure hemp oil during storage, with denser 2% agarose matrices providing enhanced volatile retention through molecular entrapment that restricts the diffusion of both oxygen and oxidation products [39].

In bigel systems, an interesting phenomenon can be observed-PV and TBARS values often show synchronous upward trends, differing from typical oxidation kinetics in simple oil systems where PV peaks then decline as primary products decompose into secondary ones [10,40]. This behaviour indicates that hydroperoxide generation rate exceeds decomposition rate during the acceleration stage, and the continuous PV increase reflects the complex, non-uniform oxidation kinetics inherent to heterogeneous bigel matrices [10], as the fatty acid composition of the oil phase fundamentally determines oxidative susceptibility. Dridi et al. (2016) found a positive correlation between the oxidation and the double bond index (R = 0.99) in W/O emulsions [41].

### 2.6. Association Colloids and Reverse Micelles

**Figure 6:**
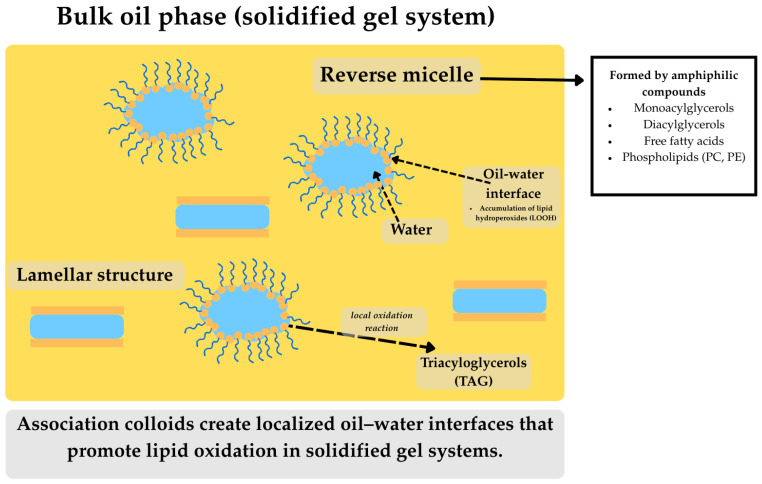
Association colloids create localised oil–water interfaces that promote lipid oxidation.

In solidified gel systems, bulk oil components (especially cold-pressed, non-refined) contain various amphiphilic compounds that can entrap water molecules, forming reverse micelles [13,19]. These structures, called association colloids, are primarily lamellar structures and reverse micelles (Figure 6). Different water qualities or concentrations of surface-active compounds can yield various shapes of association colloids and, thus, affect lipid oxidation in different ways [13,27]. Some surface-active compounds have low hydrophilic–lipophilic balance values, namely monoacylglycerol (≈3.4–3.8), diacylglycerol (≈1.8), and free fatty acids (≈1), which makes them form reverse micelles in bulk oil, while phospholipids with a medium hydrophilic–lipophilic balance value (≈8) create reverse micelles and lamellar structures in bulk oil. Lipid hydroperoxides can also reportedly accumulate at the interfacial region of reverse micelles [13,19].

Association colloids can affect oxidation rates by forming oil–water interfaces where antioxidants and pro-oxidant compounds interact with triacylglycerols [13,16,19]. Phosphatidylcholine (PC) and phosphatidylethanolamine (PE) act as pro-oxidants in striped oils when their concentrations exceed the critical micelle concentration (CMC). The pro-oxidant activity is attributed to oil–water interfaces formed by phospholipid reverse micelles, which alter the location of amphiphilic and hydrophilic pro-oxidant compounds, thereby bringing them closer to the triacylglycerol substrate. In addition, phospholipids can decrease oil surface tension, thereby enhancing oxygen diffusion in the oil [13,34]. Meanwhile, free fatty acids affect the reverse micelle structure of 1,2-dioleoyl-sn-glycero-3-phosphocholine (DOPC) in bulk oil, exhibiting pro-oxidant effects by binding metal ions, rendering them more pro-oxidative, and accelerating the decomposition of lipid hydroperoxides [34].

### 2.7. Impact of Gel Structure on Oxidation Kinetics

The three-dimensional network of bigels and emulgels impacts oxidation kinetics through physical barriers and microenvironmental effects [10,11,42]. Crystal morphology in oleogel phases affects oxygen diffusion and lipid accessibility, as confirmed by studies on candelilla wax and rice bran oil oleogels, which showed that dense, small-crystal networks with high surface area provide better protection against oxidation than large, loosely packed crystals [2,42]. The crystal form also matters, with β-polymorphs generally offering superior stability compared to α-forms due to tighter molecular packing.

The crystalline structure of the oleogel phase plays a pivotal role in oxidative stability by affecting lipid-free radical transfer processes [10,11]. The increases in dense crystal networks in the oleogel phase in bigels can inhibit lipid oxidation by physically restricting radical mobility and oxygen diffusion [10,30]. However, thermal processing that disrupts crystalline organisation—as occurs when beeswax oleogel transitions from gel to sol state at elevated temperatures—increases interfacial area and the mass transfer of oxidants, accelerating oxidative degradation. X-ray diffraction (XRD) analysis demonstrated that emulsifier addition disrupted the molecular order of crystal structures (increased full width at half maximum from 2.288° to 2.655–3.482°), yet the emulsifier-mediated network at low concentrations (0.5–1% glyceryl monostearate-GM) facilitated stronger coupling between oleogel matrix and hydrogel particles, creating a synergistic composite structure with enhanced elasticity [11].

The hydrogel-to-oleogel ratio in bigels balances physical protection and pro-oxidant exposure [13,43,44]. Emulgels containing linseed oil and sunflower oil exhibited higher resistance to peroxidation than emulsions containing these oils, attributed to the limited movement of pro-oxidants in the aqueous phase toward unsaturated lipids in the oil phase, resulting in a lower peroxidation rate of emulgel [13,30]. Higher gelatin content in bigels correlated with greater oxidation susceptibility, likely due to thermal degradation of gelatin during processing and consequent weakening of the structural barrier [43]. In bigel-based oil spreads, structuring with gelatin or agar significantly reduced the rate of primary oxidation product accumulation compared to pure oil, where peroxide values exceeded 10 mEq/kg after only 7 days of storage, followed by rapid acceleration between days 14 and 30 [45].

### 2.8. Thermal Processing Impact on Oxidation

Thermal processing is a significant factor in the destabilisation of structured lipids, as elevated temperatures accelerate both enzymatic and non-enzymatic oxidation pathways [46,47,48]. In oil blends with a 5:1 ratio of omega-6 to omega-3 fatty acids, the heating at 170 °C and 200 °C resulted in substantial losses of tocopherols, with the total content reduced to approximately 16% of the original content in unheated samples [46]. However, oil blends incorporating wheat germ oil, which contains higher levels of all tocopherol forms, showed significantly better oxidative stability during thermal treatment, with reduced formation of polar compounds and triacylglycerol polymers [46,49].

The formation of polar compounds, which serve as an indicator of extensive oxidation, increased on average 1.9-fold at 170 °C and 3.1-fold at 200 °C compared to unheated oils [46,50]. The thermal decomposition of bigels and emulgels occurs already at approximately 50 °C [3], significantly lower than the melting points of conventional fats, suggesting that these systems are primarily suitable for cold or mildly heated applications rather than high-temperature cooking processes [3,51,52]. Under accelerated oxidation conditions at elevated temperatures (50 °C), W/O bigels containing beeswax oleogel showed significantly higher peroxide values than O/W systems (*p* < 0.05), indicating that oxidative stability under thermal stress is dominated by oleogel phase behaviour [44].

## 3. Mechanisms of Fat Mimicry and Structural Properties

### 3.1. Oil-Binding Capacity and Oleogel Formation

A critical determinant of the efficacy of structured lipids as fat replacers is oil-binding capacity (OBC), which directly influences oxidative stability by controlling oil release and interfacial exposure. In sunflower oil-based oleogels enriched with phenolic extracts, the OBC remained at 87.45% when enhanced with natural antioxidants, compared to 95.17% in unmodified oleogels [2]. The only slight reduction in OBC with maintained structural integrity offers a compromise between oxidative protection and functional performance. The binding mechanism in such oleogels operates through the crystallisation of lipophilic gelators, such as beeswax and glycerol monostearate, which form a three-dimensional network capable of entrapping liquid oil within its matrix [1].

The gelation process is ruled by the molecular structure of the gelator and its ability to form stable crystal networks. In potato starch-glycerol monostearate bigels, the hydrogel hardness (1423.47 g) directly influences the overall viscosity and firmness of the bigel system, with hydrogel-dominant formulations (80% hydrogel phase) exhibiting higher viscosity and greater structural firmness [3]. Furthermore, the oleogel-to-hydrogel ratio determines the thermal stability and plasticity of the final product; systems with higher oleogel content exhibit superior thermal stability, a softer texture, and higher yield stress, indicative of optimal spreadability [3]. The oil-binding capacity directly impacts oxidative stability by determining the extent of oil exposure at interfaces where oxidation preferentially occurs.

### 3.2. Phase Ratio Effects on Physical-Rheological Properties

**Figure 7:**
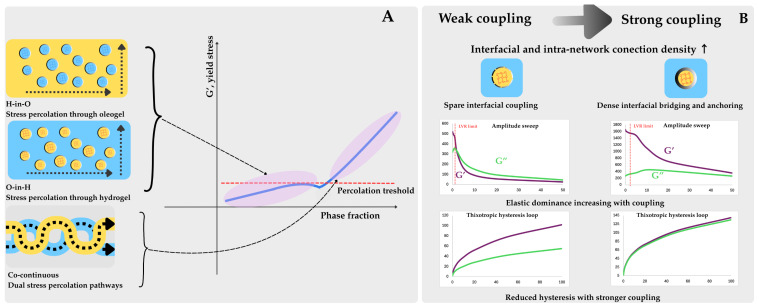
(**A**) Schematic illustration of stress percolation pathways and the percolation threshold in different bigel morphologies. (**B**) Schematic rheological responses of weakly and strongly coupled bigels, illustrating elastic dominance and changes in thixotropic hysteresis; the curves are schematic and represent estimated qualitative trends.

Manipulating the oleogel-to-hydrogel ratio enables the development of tailor-made bigel systems with predetermined physical and rheological characteristics that influence oxidation susceptibility (Figure 7).

Barroso et al., observed that bigels exhibit shear-thinning and thermal sensitivity at ~50 °C [3]. This thermal limitation defines the applicability window for bigel-based fat replacers, restricting them to cold or mildly heated applications where structural integrity and, consequently, oxidative protection can be maintained.

The storage stability of bigels over a 21-day period showed that systems with higher hydrogel phases were less stable, confirming that moisture-holding capacity can paradoxically promote phase separation when excessive [3]. Phase separation exposes previously protected oil surfaces to pro-oxidant species, making phase stability a prerequisite for sustained oxidative protection. The critical importance of optimising the hydrophilic-to-lipophilic phase ratio extends beyond physical stability to direct oxidative consequences: excessive aqueous phase provides a reservoir of dissolved oxygen and metal ions, while insufficient aqueous phase may limit the structural barrier function of the hydrogel network [13,43,44]. In emulgels stabilised with multilayered polysaccharide–chitosan interfaces, the charge reversal from −32.4 mV to +38.0 mV demonstrated successful formation of stabilising layers around oil droplets, enhancing both colloidal and oxidative stability [53]. The presence of such encapsulating materials creates a barrier effect that shields encapsulated bioactive compounds and lipids from degradation, thereby extending shelf-life.

**Figure 8:**
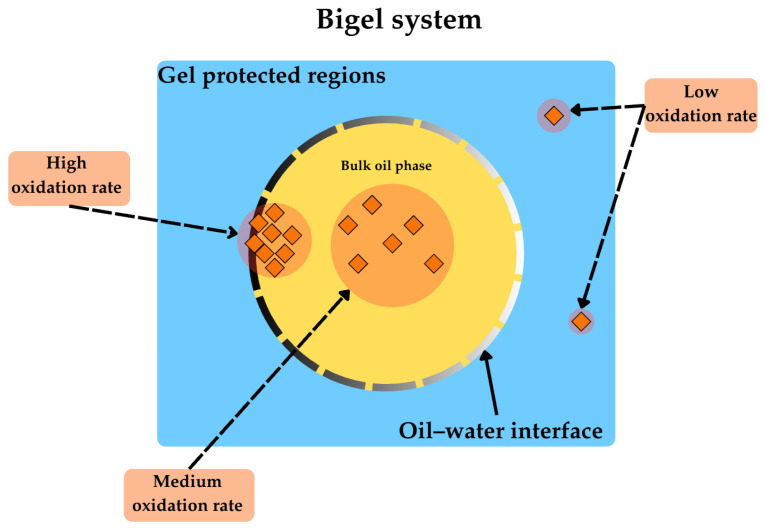
Oxidation phenomena in pure phases comparing to interphase.

Lipid oxidation in bigels is multifactorially dependent on the system structure (oleogel-in-hydrogel, hydrogel-in-oleogel, or bicontinuous), the type of oleogel, thermal processing conditions, and the presence of natural antioxidants [9]. Unlike simple oil systems where oxidation kinetics follow predictable patterns, bigels exhibit rare oxidative behaviours arising from the non-continuous biphasic nature and the critical role of the oil–water interface as a reactive zone. The oxidation reaction in bigels occurs preferentially at the oil–water interface, whereas in the bulk oil phase or in areas protected by the gel network, oxidation proceeds more slowly [10] (Figure 8).

## 4. Pro- and Anti-Oxidant Activity of Basic Ingredients

**Figure 9:**
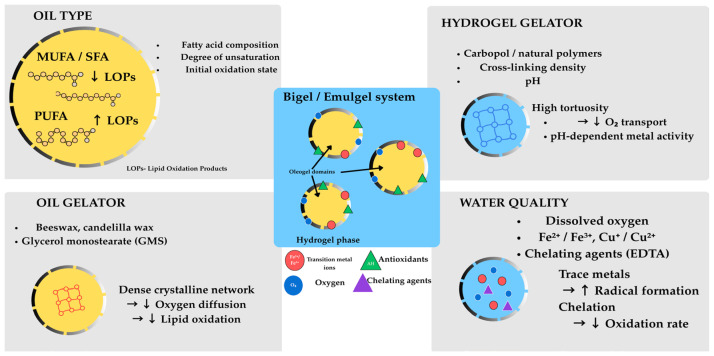
Key factors controlling oxidative stability in bigel and emulgel systems.

Solidified fat in the form of bigels and emulgels forms the matrix of ingredients that act simultaneously pro- and anti-oxidative (Figure 9).

### 4.1. Oil Type and Characteristics

The inherent susceptibility of oils to oxidation in bigels and emulgels is predominantly determined by their fatty acid composition, particularly the degree of unsaturation [13,16,47]. While the relationship between fatty acid unsaturation and oxidation susceptibility is well established for bulk oils and fluid emulsions, the following discussion addresses how these relationships are modulated by the biphasic gel architecture of bigels and emulgels, where interfacial area, phase viscosity, and oil droplet confinement alter the effective oxidation kinetics of each oil type. Polyunsaturated fatty acid (PUFA)-rich oils, such as soybean oil containing approximately 7% linolenic acid, generate significantly higher concentrations of lipid oxidation products (LOPs) compared to monounsaturated fatty acid (MUFA)-rich oils when exposed to thermal stress or oxygen [47,50]. During heating at 100–200 °C, soybean oil showed the highest formation of secondary oxidation products, including α,β-unsaturated aldehydes, with increases exceeding 2000% in certain volatile aldehydes, such as 2,4-heptadienal [47,54]. The initial oxidation state of oils before bigel formation also matters significantly; oils with pre-existing primary oxidation products (lipid hydroperoxides) will accelerate the formation of secondary oxidation products during processing and storage [40,55].

Saturated and monounsaturated oils demonstrate superior oxidative stability due to the absence of bis-allylic hydrogen atoms that are particularly susceptible to hydrogen abstraction by reactive radicals [16,19]. The selection of oil type determines the baseline oxidative stability of the resulting bigels or emulgels, with oleic acid-rich oils and palm oil demonstrating greater resistance to peroxidation than PUFA-rich alternatives [15,56].

The viscosity and polarity of the oil phase also influence oxidation kinetics by affecting the molecular mobility of substrates and pro-oxidants within the oleogel matrix [13,18,26]. Oils with higher viscosity may somewhat restrict oxygen diffusion and radical mobility, potentially slowing oxidation rates, though this effect is typically minor compared to the effects of fatty acid composition [15,30]. Additionally, the presence of endogenous minor components in oils—such as trace metal ions, tocopherols, and other natural antioxidants—contributes substantially to the oil’s inherent antioxidant capacity [34,42,49].

### 4.2. Hydrogel Gelator Type and Characteristics

The hydrogel phase both physically constrains the oleogel phase and potentially introduces antioxidant or pro-oxidant species [9,11,57]. Beyond their well-documented role as structural scaffolds, hydrogel gelators in bigels and emulgels simultaneously function as diffusion barriers, metal-ion reservoirs, and pH-buffering matrices—properties that collectively modulate oxidative stability in ways absent from single-phase gel systems. Carbopol 940, a frequently used hydrogel gelator, forms a three-dimensional cross-linked polymer network restricting the diffusion of molecular oxygen and free radicals into the oil phase [57,58,59]. The degree of cross-linking in the hydrogel determines the tortuosity of diffusion pathways, thereby influencing the oxygen availability for lipid oxidation [54,55]. Natural polymer-based hydrogels, such as guar gum and cellulose, introduce additional complexity due to their inherent water-binding capacity and chemical interactions with metal ions and antioxidants [44,51,59]. The polymer structure affects not only the rheological properties but also the distribution and accessibility of water molecules, which is connected with metal ion concentration and activity in the aqueous phase [9,13].

The pH of the hydrogel phase is a critical parameter because it modulates the ionisation state of chelating agents and affects the redox potential of metal ions [16,31,32]. At neutral to slightly acidic pH values maintained in many bigel formulations, transition metals like iron and copper are more likely to exist in oxidation states that actively catalyze free radical generation [32,33]. Additionally, polymeric gelators contain residual ionic or non-ionic impurities from synthesis, which may either enhance or diminish oxidative stability [58,60]. Carbopol and other acrylic acid-based polymers, for instance, contain free carboxylic acid groups that could potentially chelate divalent metal ions and reduce their pro-oxidant activity, though this effect has not been extensively characterised in the context of bigel systems [57,59].

### 4.3. Oil Gelator Type and Characteristics

In bigels and emulgels, oil gelators serve a dual function: they determine the physical state of the oleogel phase, but they also define the crystalline barrier properties that control oxygen permeation and radical mobility across the interphase—a role with no equivalent in bulk oil or fluid emulsion systems. Oil gelators such as beeswax, candelilla wax, and Span surfactants control the physical state of the oleogel phase and determine the crystalline or amorphous network structure that constrains the liquid oil [2,9,42]. The gelator type influences the permeability of the oleogel network to oxygen and small molecular species [10,21,61]. Oleogels prepared with beeswax demonstrated reduced lipid oxidation compared to lower-melting-point gelators, potentially due to their denser crystalline structure that limits oxygen diffusion [2,44,62].

Glycerol monostearate (GM), when used as a gelator or co-gelator, can strengthen the oleogel structure through favourable interactions with wax molecules, thereby potentially decreasing oxygen accessibility to the oil phase [11,42,61]. The gelator’s thermal stability is also critical—gelators that undergo phase transitions or decomposition during processing or storage can generate degradation products with pro-oxidant or antioxidant properties [10,60]. Additionally, the surface characteristics of the gelator crystals influence their interaction with the aqueous phase at the interphase, affecting water uptake, metal ion accumulation, and the distribution of emulsifiers or antioxidant compounds [11,15,61].

### 4.4. Water Quality and Its Impact on Oxidation

Water quality strongly modulates oxidative stability in bigels and emulgels through dissolved ions, dissolved oxygen, and trace metal contamination [16,32,63]. Water quality is a frequently underestimated parameter in biphasic gel systems, where the aqueous phase is not merely a diluent but an active compartment delivering dissolved oxygen, catalytic metal ions, and chelating species directly to the oil–water interphase—the primary site of oxidation initiation. The concentration of dissolved oxygen in the aqueous phase is directly proportional to the rate of lipid oxidation in adjacent oleogel domains, as oxygen must diffuse through the hydrogel matrix to reach the oil phase [11,13]. Similarly, the dissolved metal ion content of the water phase—particularly iron (Fe^2+^/Fe^3+^) and copper (Cu^2+^/Cu^+^)—determines the catalytic activity of the system [31,32,33]. Water purification processes such as reverse osmosis or distillation reduce metal ion contamination improving oxidative stability. However, even in highly purified water, trace metal ions derived from processing equipment, storage containers, or residual impurities in gelators can accumulate during manufacturing and storage [63,64].

The presence of chelating agents or chelating capacity in the aqueous is a control parameter [31,32]. Water systems treated with ethylenediaminetetraacetic acid (EDTA) or other chelators can effectively suppress metal-ion-dependent lipid oxidation [63,65,66]. However, the effectiveness of chelation depends on pH and the specific ligand properties—for instance, EDTA chelation complexes with transition metals can remain partially redox-active and facilitate oxidation under certain conditions [38,63]. Ultrapure or deionized water initially presents lower oxidative risk, but during the storage and processing of bigels, it may accumulate metal ion contaminants from the polymer gelators, oils, or container leaching [31,64]. The buffering capacity and pH of the aqueous phase influence metal speciation and, therefore, their redox activity; at higher pH values (typically 7–8), metal ions are more likely to exist in less reactive forms or form precipitates [13,32,34]. Additionally, the osmotic strength of the aqueous phase, depending on dissolved salts and other solutes, influences both water migration between phases and the activity coefficients of dissolved ions [35,67].

## 5. Processing-Induced Oxidation

The preparation process itself can initiate or accelerate lipid oxidation, particularly when high-temperature gelation is required [5,40,47]. Ethylcellulose-based oleogels, which require processing temperatures around 145 °C for complete polymer dissolution, provide the most notable example [5,44]. This thermal treatment promotes free radical formation and liberation of unsaturated fatty acids, subsequently initiating chain reactions and accelerating hydroperoxide decomposition into secondary oxidation products [16,47,50].

The consequences of thermal processing have been clearly demonstrated in meat applications. When animal fat was replaced with ethylcellulose oleogel-based bigel in beef burgers, TBARS values increased significantly throughout frozen storage, reaching approximately 1.3–1.4 mg MDA/kg at 75% fat replacement compared to 0.4 mg MDA/kg in control samples—representing a 3-fold increase in oxidation products [5,9]. Additionally, emulsion production enhanced oxidation through oxygen incorporation, acoustic cavitation during sonication, or overheating from shear stress [13,15].

Interfacial engineering through emulsifier can modulate interface-mediated oxidation [11,16,34]. Studies with glyceryl monostearate (GM) and phosphatidylcholine (PC) revealed that both emulsifiers effectively inhibited lipid oxidation after 21 days of storage by significantly reducing oil–water interfacial tension from 18.10 to 12.90 mN/m [11,68]. GM-containing bigels showed peroxide value reductions of 64–69%, while PC-containing bigels demonstrated reductions of 41–60% [11]. The protective mechanism involves dual inhibitory functions: slowing the migration of water-soluble pro-oxidants from the hydrogel phase into the continuous oil phase, and limiting amphiphilic hydroperoxide access to the high-water-activity interface [11,19,27].

### 5.1. Temperature Effects

Temperature is a crucial variable for oxidation rates in bigels and emulgels [10,40,47]. The formation of lipid oxidation products shows an Arrhenius-type temperature dependence, with oxidation rates typically doubling or tripling for every 10 °C increase within the 20–60 °C range [47,49]. At elevated processing temperatures, lipid peroxidation dramatically accelerates—heating at 200 °C generates oxidation products at rates thousands of times higher than at room temperature, with PUFA-rich oils particularly vulnerable [47,50,54]. The apparent activation energy for lipid oxidation varies between 28–73 kJ/mol depending on oil type and fatty acid composition [18,40].

Moderate temperature increases during processing can sometimes improve bigel stability by promoting uniform gelator distribution or enhancing crystalline network formation [5,10,61]. Storage temperature is equally critical—maintaining bigels at 4 °C or below substantially delays oxidation by reducing molecular mobility and slowing diffusion-limited steps [44,63,69]. Temperature fluctuations during storage dramatically accelerate lipid oxidation and promote water migration between phases, further destabilising bigel/emulgel structure [70,71].

### 5.2. Processing Speed and Mechanical Shear

The speed of mixing, homogenization, or mechanical processing during bigel preparation influences oxidative stability through multiple mechanisms [13,15,72]. High-speed mixing increases the surface area between the aqueous and oleogel phases by promoting emulsification and reducing droplet size, increasing interfacial area for oxygen transfer [11,15]. Mechanical shear is a source of frictional heat, locally elevating temperatures and potentially accelerating oxidation [5,61]. Additionally, shear rate influences antioxidant distribution—insufficient mixing results in a heterogeneous distribution, with certain regions remaining unprotected [9,10]. Intense processing can partially disrupt the protective oleogel network structure or cause phase inversion, particularly in bigels with high oleogel concentrations [11,44].

The optimal processing speed represents a balance between achieving adequate homogeneity and minimizing reactive species generation [5,72]. Extended processing times subject the formulation to prolonged exposure to dissolved oxygen and elevated temperatures, thereby progressively increasing oxidative damage [13,16].

### 5.3. Atmospheric Composition and Oxygen Availability

Atmospheric oxygen availability is an absolute requirement for lipid peroxidation to occur at meaningful rates [10,16,69]. Processing and storage under inert gas atmospheres (nitrogen or argon) substantially reduce oxidation rates, though complete prevention is impossible due to oxygen dissolved in oil during initial mixing or trapped within the gel matrix [9,16]. Vacuum packaging or nitrogen flushing can also extend shelf-life by reducing available oxygen [69,71]. In W/O bigels where water is dispersed within oleogel, dissolved oxygen in both phases becomes relevant—water typically contains approximately 8–9 mg/L dissolved oxygen at room temperature, serving as a local source for oxidation reactions even when the external atmosphere is nitrogen-purged [10,13,73].

### 5.4. Light Exposure

Light exposure, particularly in the ultraviolet and visible regions below 500 nm, photosensitizes lipid oxidation through multiple mechanisms [16,18,74]. Direct photolysis of dissolved oxygen can generate singlet oxygen (_1_O_2_), an extremely reactive species that oxidizes unsaturated fatty acids at rates approximately one thousand times faster than ground-state triplet oxygen [18,75]. Photosensitized oxidation can be initiated by natural pigments in oil (chlorophyll, carotenoids) or introduced photosensitizers [16,76]. Additionally, light promotes the thermal decomposition of existing lipid hydroperoxides into highly reactive alkoxyl and peroxyl radicals, which further propagate chain reactions [19,30]. Protection from light through amber or opaque packaging substantially reduces photooxidative damage [9,69].

### 5.5. Indirect Oleogelation: The Emulsion-Template Approach and Oxidation Implications

The preceding sections focused on oleogel phases produced through direct structuring, in which lipophilic gelators such as waxes, glycerol monostearate, or ethylcellulose are dissolved in heated oil and form a three-dimensional network upon cooling [2,5,9,42]. These methods frequently require elevated temperatures—ethylcellulose-based oleogels require ~145 °C for polymer dissolution, while wax-based systems typically require 75–140 °C [5,11,44]. As discussed in Section 2.8 and Section 4.1, such thermal exposure accelerates lipid oxidation by promoting free radical formation, tocopherol degradation, and hydroperoxide decomposition into secondary products [16,46,47,50].

An increasingly important alternative—indirect oleogelation via the emulsion-template method—proceeds through a fundamentally different sequence. An O/W emulsion is prepared using food-grade biopolymers (proteins, polysaccharides, or their electrostatic complexes) as emulsifiers [23,24,77]. These biopolymers adsorb to oil droplet surfaces forming a protective interfacial layer. The aqueous phase is then removed by freeze-drying or mild convective drying below 70 °C, causing biopolymer-coated droplets to aggregate into a continuous network entrapping liquid oil [24,78]. The resulting oleogel (90–98% oil content) derives structural integrity from the biopolymer shell network rather than crystalline mechanisms [9,79].

From an oxidation perspective, the primary advantage is avoidance of high-temperature processing. Whereas ethylcellulose oleogels require ~145 °C—conditions promoting substantial tocopherol losses (reduced to ~16% of original content at 170 °C [46]), accelerated polar compound formation (1.9-fold increase at 170 °C [46,50]), and free radical chain initiation [16,47]—the emulsion-template approach conducts emulsification below 60 °C, with subsequent drying well below significant thermal oxidation thresholds [24]. Freeze-drying operates under vacuum at sub-zero temperatures, minimising both thermal and oxygen-mediated oxidation [78].

Additionally, the biopolymer gelators used are predominantly GRAS-status, relevant for clean-label food formulations [9,77]. Certain biopolymer emulsifiers confer intrinsic antioxidant activity: protein-based interfacial films scavenge free radicals through amino acid residues (tryptophan, tyrosine, cysteine), while polysaccharides such as chitosan exhibit metal-chelating properties [13,16,80]. Emulsion-templated oleogels prepared with pectin–low-density lipoprotein complexes significantly suppressed both primary and secondary lipid oxidation in PUFA-rich systems [77]. Oleogels structured with whey protein concentrate–xanthan gum or whey protein concentrate–basil seed gum complexes showed improved oxidative stability, with centrifuge stability increasing from 26% (protein alone) to nearly 100% with hydrocolloid incorporation [23].

However, the emulsion-template approach introduces its own oxidation challenges. During emulsification, oil is dispersed into fine droplets with large total interfacial area, creating extensive oil–water contact zones where pro-oxidant species access lipid substrates—the same interfacial mechanism discussed in Section 2.3 and Section 2.4 [13,15,16,32]. During drying, water removal progressively concentrates pro-oxidant species in the diminishing aqueous phase, potentially accelerating interfacial oxidation [24]. Incomplete drying may leave residual water pockets serving as localised loci for metal-ion-catalysed oxidation [31,32,63]. Freeze-drying, while thermally gentle, creates highly porous structures with large internal surface area exposed to atmospheric oxygen during storage [9,16,69].

When emulsion-templated oleogels are incorporated into bigel systems, the reintroduction of an aqueous phase re-establishes oil–water interfaces absent in the dried oleogel. Whether the original biopolymer coating remains intact after mechanical mixing, or is disrupted exposing fresh oil surfaces to pro-oxidant species, remains largely unexplored and represents a significant research gap [11,27].

In summary, the emulsion-template approach reduces processing-induced oxidation principally by avoiding high temperatures required for direct oleogelation, but introduces oxidation risks during emulsification, drying, and bigel assembly that require careful formulation design—including selection of interfacial biopolymers with antioxidant functionality, minimisation of holding times between processing steps, and appropriate packaging to limit post-processing oxygen exposure.

## 6. Oxidative Stability Assessment Methods

Oxidative stability, the most crucial parameter in fat-mimicking structures, requires a robust analytical methodology. Research shows that multiple methods are most commonly used by researchers, providing a foundation for a standard for the quality analysis of these structures that may serve as a basis for future quality norms in industrial production.

### 6.1. Limitations of Oxidation Measurement in Bigel and Emulgel Structures

Several methodological challenges complicate oxidation assessment in bigels and emulgels [9,10,15,16]. The oil extraction step required for most analyses may introduce artefacts, as centrifugation and vortexing expose recovered lipids to oxygen and heat, potentially accelerating oxidation or modifying the product profile [10,14,44].

The biphasic nature means oxidation products partition differently between phases, and standard procedures developed for bulk oils may not efficiently recover all products from the gel matrix [13,15,16]. Furthermore, in rapidly oxidising systems such as marine phospholipids, hydroperoxide and aldehyde formation can proceed too fast for detection by PV, AnV, or TBARS, leading to erroneous conclusions of no oxidation [16].

Conversely, the gel matrix may bind secondary products through Maillard-type reactions with proteins or polysaccharides, underestimating oxidation extent [9,13,16], while TBARS assays overestimate malondialdehyde due to interference from hydrogel-phase sugars and amino acids [10,69].

Spatial heterogeneity presents an additional challenge: oxidation proceeds at different rates at interfaces, within oil droplets, and in the continuous phase, but bulk methods average across these microenvironments, masking localised patterns [15,16]. Spatially resolved techniques such as confocal Raman microscopy or imaging mass spectrometry can address this limitation [12,46], and standardised protocols specifically designed for structured gel systems are needed to enable meaningful cross-study comparisons [9,13,15,16].

### 6.2. Analytical Methods for Oxidation Assessment in Structured Lipid Systems

The principal methods employed for oxidation assessment in bigels and emulgels are summarised in Table 1, which maps each technique to its specific application in gel-based systems and its associated limitations.

Rather than recapitulating standard lipid oxidation methodology—comprehensively reviewed elsewhere [13,14,16,81]—the following overview focuses on how these methods perform in the context of structured biphasic matrices and what system-specific behaviours they reveal.

Primary oxidation is assessed by peroxide value (PV) assays that quantify lipid hydroperoxides, most commonly via ferrous iron/thiocyanate colourimetry at 510 nm [10,14,44,81]. In bigel systems, PV monitoring has revealed oxidation kinetics that differ fundamentally from those in bulk oils: PVs increase continuously throughout storage rather than peaking and declining, reflecting the non-uniform, spatially heterogeneous progression of oxidation across different microenvironments within the gel matrix [10]. In O/W bigels without added antioxidants, PV reached 107.2 mmol/kg oil after 35 days at 25 °C [10], while in bigel-based oil spreads structured with gelatin or agar, gelation significantly reduced the rate of hydroperoxide accumulation compared to pure oil, where PV exceeded 10 mEq/kg after only 7 days [45]. These contrasting values illustrate that the structural configuration—not merely oil composition—governs primary oxidation product formation rates.

Secondary oxidation is tracked through thiobarbituric acid reactive substances (TBARS) for malondialdehyde, p-anisidine value (AnV) for aldehydes, and the combined TOTOX index (2 × PV + AnV) [10,11,12,17]. A characteristic feature of bigel systems is the hysteresis effect in TBARS curves: values remain low during the first 14 days of storage, then increase sharply as accumulated hydroperoxides decompose into secondary products [10]. Notably, PV and TBARS values show synchronous upward trends in bigels—a behaviour absent in homogeneous oil systems, where PV typically peaks before declining as secondary products form [10,15]. This synchronous increase indicates that hydroperoxide generation rate exceeds decomposition rate during the acceleration stage, and that overlapping oxidation stages in distinct microenvironments within the bigel produce complex, non-uniform apparent kinetics [9,10,16]. Comparative studies confirm that W/O structures generally exhibit lower TOTOX values than O/W systems, consistent with reduced interfacial area and restricted oxygen access [9,13,15].

Advanced instrumental techniques provide mechanistic insights beyond what bulk extraction methods can reveal. GC-MS with headspace SPME identifies specific volatile oxidation markers (hexanal, pentanal, acetone from epidioxide decomposition) and enables attribution of off-flavour development to particular degradation pathways [13,16]. FTIR spectroscopy offers non-destructive, real-time monitoring of functional group changes—particularly cis double bond disappearance and carbonyl group formation—and has been applied to characterise emulsifier–matrix interactions in bigels, where O–H stretching vibration peaks (3472–3476 cm^−1^) showed no significant shift upon GM or PC addition, indicating weak hydrogen bonding between emulsifiers and the bigel matrix [11]. DSC measures oxidation induction time under accelerated conditions, enabling comparative stability ranking across formulations [5,9,61]. XRD tracks crystal structure changes in oleogel phases during oxidation and storage, with emulsifier-induced broadening of full width at half maximum values (from 2.288° to 2.655–3.482°) correlating with altered physical barrier properties [11].

CLSM enables visualisation of phase distribution and droplet morphology through fluorescent staining of oil and aqueous phases, providing direct observation of the microstructural features that govern interfacial oxidation exposure [15,44,61]. Recent work has demonstrated the utility of electronic nose technology (PEN3 system) for monitoring oxidation-related volatile changes in structured lipid systems, where sensor responses tracking sulfur-containing VOCs revealed progressive lipid oxidation during storage of agarose-structured hemp oil, with pure oil showing a 49% decrease in desirable volatile retention compared to structured systems [31]. NMR and SAXS provide complementary information on molecular dynamics, water and oil phase mobility, and nanoscale structural organisation, though their application to oxidation monitoring in bigels remains limited [9,16].

## 7. Antioxidant Strategies and Protective Solutions

### 7.1. Natural Antioxidant Integration and Phenolic Compounds

Natural antioxidants are the most effective and consumer-accepted approach for controlling lipid oxidation in bigel systems [9,13,16]. High-performance liquid chromatography (HPLC) analysis identified quercetin (432.15 μg/g) and kaempferol (295.23 μg/g) as the predominant phenolic compounds in onion peel extract used for emulgel enrichment [2]. The total phenolic content (TPC) of enriched emulgels reached 5.49 mg GAE/g with an antioxidant activity of 38.84% (DPPH assay), indicating substantial protective capacity against lipid oxidation. Research has conclusively demonstrated that bigels containing antioxidants showed significantly reduced PV and TBARS values [10,44,45].

Rasekhi Kazeruni et al. (2025) investigated ferulic acid and γ-oryzanol in emulsion and emulgel based on black seed oil [14]. In the initiation phase, ferulic acid at 2.32 mM concentration showed the highest efficiency in emulgel samples, whereas γ-oryzanol at 2.32 mM concentration showed the highest efficiency in emulsion samples. γ-Oryzanol was more effective than ferulic acid in emulsion and emulgel samples in the propagation phase. The γ-oryzanol and ferulic acid participated in side reactions of initiation chain in addition to participating in the major termination reaction [5,14]. Tocopherols, particularly α-tocopherol, function as chain-breaking antioxidants by donating hydrogen atoms to lipid peroxyl radicals, converting them into stable hydroperoxides while forming tocopheroxyl radicals [16,60].

### 7.2. Synergistic and Antagonistic Antioxidant Combinations

Synergistic combinations of hydrophilic and lipophilic antioxidants creates oxidation system control in bigel systems [10,16]. The co-encapsulation of astaxanthin (AST) and ascorbic acid (AA) demonstrated exceptional antioxidant synergy, with interaction indices (Q) greater than 1 across all bigel types: 1.31 in O/W bigels, 1.24 in bicontinuous bigels, and 1.07 in W/O bigels [10]. The complementary mechanisms was involved: AA scavenges free radicals through reduction reactions and is oxidized to dehydroascorbic acid, while AST can regenerate AA by reducing dehydroascorbic acid and restoring its antioxidant capacity. The intermolecular attraction causes AA to rapidly transfer to the oil–water interface and oil phase, resulting in antioxidant accumulation at the interface where lipid oxidation proceeds most rapidly [10,13].

In contrast, not all antioxidant combinations yield synergistic effects. Curcumin combined with AA showed antagonistic interactions (Q < 1) with values of 0.92, 0.80, and 0.87 in O/W, bicontinuous, and W/O bigels, respectively [17]. Similarly, resveratrol and AA demonstrated antagonism with Q values of 0.69, 0.74, and 0.75 across the three bigel types. This antagonism may result from competition in free radical scavenging processes, reducing overall antioxidative effect, or mutual interference affecting distribution at the oil–water interface. Since resveratrol and curcumin are both polyphenolic compounds with multiple phenolic hydroxyl groups, these functional groups may interact with the reducing capacity of AA during oxidative processes [10,16].

### 7.3. Plant-Derived Phenolic Extracts and Fruit Pomace

Plant-derived phenolic extracts have wide applications in food products [9,16,69]. Rosemary extract (RE) containing rosmarinic acid, carnosol, and carnosic acid significantly enhanced the antioxidant effect of bigel coatings on sardine fillets stored at 4 °C [69]. The carnosic acid and carnosol typically found in rosemary extracts protect against oxidation progress by stabilizing hydroperoxides and inhibiting their decomposition into active forms such as malonaldehyde, while also creating complexes with Fe^2+^ to prevent hydroxyl radical formation [69]. The incorporation of 2% RE into bigel coatings produced significantly lower TBARS (*p* < 0.05) compared to both uncoated controls and coatings without RE. After 4 days of storage, TVB-N values were 7.7 mg/100 g in bigel coatings with RE incorporated in the oleogel phase (BGOR) and 10.5 mg/100 g with RE in the hydrogel phase (BGHR), compared to 26.6 mg/100 g in uncoated control samples—representing reductions of 71% and 61%, respectively [9,69].

Fruit pomace incorporation represents another effective strategy [9,45]. The addition of lingonberry pomace to bigel-based vegetable oil spreads significantly enhanced their oxidative stability [45]. Final peroxide values were 14.22 ± 0.86 and 15.55 ± 1.82 mEq/kg in pomace-containing bigels, substantially lower than in corresponding systems without pomace. Phenolic compounds present in lingonberry pomace (TPC 6.26 GAE/g d.m.) are characterized by antioxidant activity due to the phenol ring which can provide hydrogen bonds from hydroxyl groups and delocalize unpaired electrons [37]. DPPH and Oxipres tests confirmed that pomace presence extended the oxidation induction period by 2.1–2.4 times and increased free radical scavenging capacity to 1.61–2.03 mg TE/g immediately after preparation [13,45].

### 7.4. Interfacial Protection and Pickering Emulsion Strategies

Pickering emulsions produced by solid particles have shown high physical and oxidative stability during storage periods [13,16]. The interfacial layer thickness of Pickering emulsions (ranging from 10 nm to 100 μm) is substantially higher than surfactant-based emulsions (1–50 nm). Pickering emulsions stabilized by silica particles showed higher oxidative stability compared to emulsions produced by Tween 20, which may be related to the higher thickness of the interfacial layer [13,16]. Furthermore, microcrystalline cellulose Pickering particles showed greater oxidation stability than modified starch, with antioxidant activity attributed to a combination of free radical scavenging and the formation of a thick coating around oil droplets [13].

Molecular dynamics simulations revealed that electrostatic energy dominated bigel component binding, while van der Waals interactions were extensive and governed structural packing [11,44]. The binding energies of GM- and PC-containing systems (−17.15 × 105 kJ/mol) were significantly lower than control systems (−9.25 × 105 kJ/mol), indicating stronger interactions between emulsifiers and the bigel matrix [11]. This concept of antioxidant-loaded Pickering particles has been applied to various combinations of carrier particles and antioxidants, with protein-based particles such as zein or gliadins combined with hydrophilic phenolics showing promise in forming composite particles through antisolvent precipitation [13,16].

Plant protein-based emulsifiers are sustainable alternatives for interfacial protection strategies. However, as reviewed by Harasym et al. (2025), protein oxidation during processing can compromise the structural integrity of interfacial films, with mildly oxidized proteins forming heterogeneous, structurally weak interfaces that provide reduced protection against lipid oxidation compared to freshly prepared protein films [80]. This indicates the need to control processing conditions to preserve protein functionality in emulsion-based systems.

### 7.5. Interfacial Interactions and Emulsifier Effects

**Figure 10:**
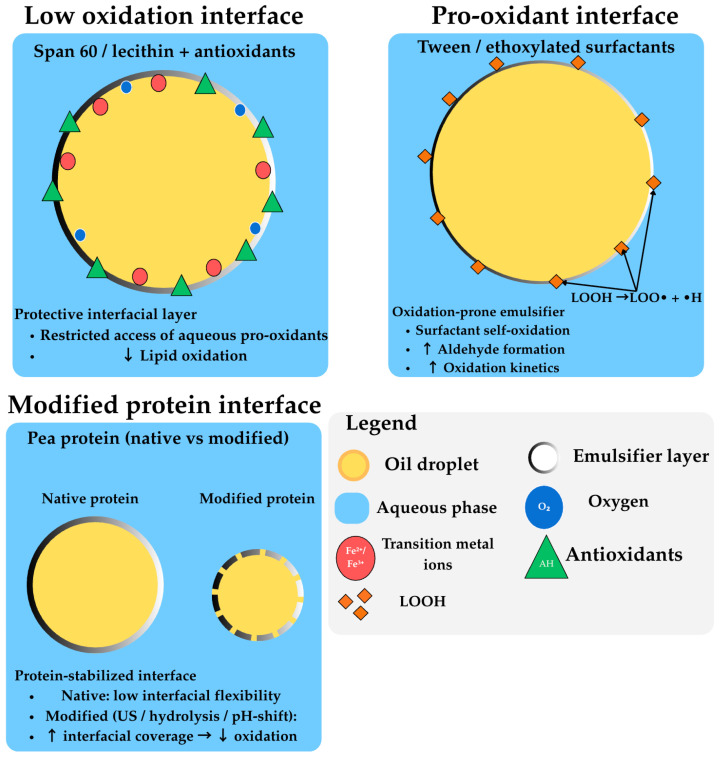
Schematic illustration of emulsifier-dependent interfacial effects on lipid oxidation in bigel and emulgel systems.

The emulsifier selected for bigel formulation significantly influences oxidative stability by affecting interfacial properties and the distribution of pro- and antioxidant species (Figure 10) [13,15,16].

The interfacial tension generated by different emulsifiers influences droplet size and distribution, which, in turn, affects the total interfacial area available for oxygen transfer and oxidative reactions. The incorporation of soybean lecithin as a co-emulsifier in caseinate-stabilized emulsions reduced lipid oxidation compared to emulsions with only caseinate [16]. Surfactant micelles may also interact with oxidation products, and depending on the charge of the surfactants, this would either favour the decomposition of hydroperoxides into more reactive radical species (acceleration of lipid oxidation kinetics) or favour the stabilisation of hydroperoxides, resulting in a delay in the formation of secondary oxidation products [15,16].

## 8. Technological Challenges and Research Gaps

Despite significant progress in characterizing oxidation in bigels and emulgels, fundamental knowledge gaps persist regarding the molecular mechanisms governing lipid oxidation at interfaces and within structured gel networks. The precise role of interfacial architecture in determining pro-oxidant and antioxidant localization remains incompletely understood, particularly in systems containing multiple emulsifiers, proteins, and polysaccharides that compete for interfacial space. Future research should employ advanced microscopy techniques (cryo-electron microscopy, confocal Raman microscopy) combined with molecular dynamics simulations to visualize antioxidant and pro-oxidant distribution at nanoscale resolution. The influence of crystal morphology and polymorphism on oxidative stability warrants deeper investigation. While studies have demonstrated that crystal type affects oxidation rates, the underlying mechanisms—whether through oxygen permeability differences, lipid accessibility variations, or altered pro-oxidant interactions—remain poorly characterized. Molecular dynamics simulations have begun to address this gap, revealing that electrostatic energy dominates bigel component binding while van der Waals interactions govern structural packing [30], but more comprehensive studies are needed to correlate molecular-level interactions with macroscopic oxidative stability.

The current thermal limitations of bigels (structural breakdown at ~50 °C) severely restrict their applicability in baking, frying, or other high-temperature applications [3]. The translation of laboratory-scale formulations to industrial production faces significant technological hurdles. High-pressure homogenization, ultrasound processing, and microfluidization offer potential for creating stable, fine-structured systems at scale, but parameter optimization specific to bigel systems remains limited. The effect of processing intensity on oxidative stability—through structural modification, temperature increases, and mechanical shear—warrants investigation to identify processing windows that maximise structural quality while minimising oxidation initiation. Furthermore, the production costs of bigels and emulgels currently exceed those of traditional fats due to multiple components and specialised processing; economic viability at an industrial scale has yet to be demonstrated.

## 9. Conclusions

The oil–water interface is the primary site governing oxidation kinetics in both systems. In emulgels, fine oil droplet dispersion creates a large interfacial area where pro-oxidant species concentrate and initiate radical chain reactions; in bigels, the interphase boundary between gel domains serves an analogous role. The composition and integrity of this interface—determined by emulsifier type, biopolymer adsorption, and processing conditions—is the single most important determinant of oxidative stability.

Structural configuration determines oxidative stability according to a consistent hierarchy: W/O > bicontinuous > O/W. This reflects reduced interfacial exposure, increased continuous-phase viscosity, and the physical barrier effects of dense oleogel networks in W/O systems, all of which restrict oxygen diffusion and pro-oxidant mobility. The gel matrix provides additional protection absent in fluid emulsions by retarding molecular transport between phases; however, this same viscosity paradoxically constrains antioxidant delivery to the interface where it is most needed.

Co-encapsulation of hydrophilic and lipophilic antioxidant pairs provide synergistic protection across both phases, while selecting emulsifiers with intrinsic barrier functionality adds a structurally integrated layer of oxidative defence. Processing conditions establish the oxidative baseline: direct oleogelation at 140–170 °C promotes antioxidant degradation and radical formation before the system is even assembled, whereas indirect emulsion-template methods achieve structuring at significantly lower temperatures, reducing processing-induced oxidation.

Based on these findings, formulators should: (i) prefer W/O or oil-continuous configurations to minimise interfacial oxidation exposure; (ii) select emulsifiers and gelators serving dual structural–antioxidant functions; (iii) deploy antioxidants as synergistic hydrophilic–lipophilic pairs positioned relative to the interfacial zone; (iv) minimise processing temperatures through indirect oleogelation and inert atmosphere conditions; and (v) complement conventional bulk-extraction analytical methods (PV, TBARS, AnV) with spatially resolved techniques suited to heterogeneous gel matrices.

Key research gaps remain. Spatially resolved oxidation mapping using confocal fluorescence microscopy or Raman micro-spectroscopy is needed to visualise oxidation initiation and propagation within these structures. Computational modelling of mass transfer and radical propagation across gel phases could provide predictive formulation tools. The oxidative behaviour of bigels incorporating emulsion-templated oleogels, the relationship between processing-induced structural changes and oxidative outcomes, and the challenges of industrial scale-up all warrant systematic investigation.

In conclusion, bigels and emulgels offer a versatile platform for healthier fat replacement, but oxidative stability remains a critical challenge that requires a mechanistically informed integration of structural design, interfacial engineering, and synergistic antioxidant strategies.

## Figures and Tables

**Table 1:** Key methods for assessing oxidative stability in gels, emulgels, and bigels.

Method Group	Method/ Index	Oxidation Products Measured	Application in Gel-Based Systems	Main Limitations	Ref.
**Primary** **oxidation**	Peroxide value (PV)	Lipid hydroperoxides	Basic method for early-stage oxidation assessment in bigels and emulgels	Requires oil extraction; no spatial information	[10,44]
	PV method (International Dairy Federation)	Lipid hydroperoxides	Standardized alternative for lipid dispersions	Limited sensitivity at advanced oxidation stages	[81]
**Secondary oxidation**	TBARS	Malondialdehyde (MDA)	Evaluation of secondary oxidation progression	Low specificity; matrix interferences	[10,44]
	p-Anisidine value (AnV)	Aldehydes	Assessment of advanced oxidation stages	Does not detect hydroperoxides	[13,15]
	TOTOX value	Primary and secondary products	Comprehensive oxidation status indicator	Indirect cumulative index	[15]
**Advanced techniques**	GC-MS/SPME-GC-MS	Volatile aldehydes and ketones	Identification of specific oxidation markers	High cost and complex sample preparation	[13,16]
	FTIR spectroscopy	Lipid functional groups	Non-destructive, real-time oxidation monitoring	Limited quantitative sensitivity	[11,44]
	DSC	Oxidation induction time	Comparative oxidative stability assessment	Accelerated conditions	[9]
**Structure-related methods**	CLSM	Microstructure and interfaces	Localization of oxidation-prone regions	Qualitative method	[15,44]

## Data Availability

No new data were created or analyzed in this study. Data sharing is not applicable to this article.

## References

[1] Quilaqueo M., Iturra N., Contardo I., Millao S., Morales E., Rubilar M. (2022). Food-Grade Bigels with Potential to Replace Saturated and Trans Fats in Cookies. Gels.

[2] Alamer H.A., Kamel R.M., Shawir S.M.S., Salama M.A., Barakat E.H. (2025). Utilizing Phenolic-Enriched Sunflower Oleogels and Emulgels as Fat Replacers: Impact on Burger Oxidative Stability, Texture, and Sensory Attributes. OCL.

[3] Alves Barroso L., Grossi Bovi Karatay G., Dupas Hubinger M. (2022). Effect of Potato Starch Hydrogel:Glycerol Monostearate Oleogel Ratio on the Physico-Rheological Properties of Bigels. Gels.

[4] Francavilla A., Corradini M.G., Joye I.J. (2023). Bigels as Delivery Systems: Potential Uses and Applicability in Food. Gels.

[5] Ghiasi F., Golmakani M.-T. (2022). Fabrication and Characterization of a Novel Biphasic System Based on Starch and Ethylcellulose as an Alternative Fat Replacer in a Model Food System. Innov. Food Sci. Emerg. Technol..

[6] Kibler N.D., Acevedo N.C., Cho K., Zuber-McQuillen E.A., Carvajal Y.B., Tarté R. (2022). Novel Biphasic Gels Can Mimic and Replace Animal Fat in Fully-Cooked Coarse-Ground Sausage. Meat Sci..

[7] Banaś K., Harasym J. (2021). Natural Gums as Oleogelators. Int. J. Mol. Sci..

[8] Abdullah, Liu L., Javed H.U., Xiao J. (2022). Engineering Emulsion Gels as Functional Colloids Emphasizing Food Applications: A Review. Front. Nutr..

[9] Farzana W., Mahesh S., Sharma S., Syed I., Abdi G., Upadhyay R. (2025). A Comprehensive Review on Bigels as a Potential Replacement to Solid Fat in Food Applications. J. Food Qual..

[10] Wei W., Guo Z., Ruzibayev A., Asliddin F., Meng Z. (2025). Bigels as Novel Co-Delivery Systems for Natural Antioxidants and Algal Oil: Oxidative Stability and in Vitro Digestive Behaviors. Food Chem..

[11] Xue Y., Zhong J., Liu X., Xiang D., Taha E., Qin X. (2026). Tuning Bigel Performance via Emulsifier Type and Concentration: Structural, Physicochemical, and Molecular Perspectives. Food Res. Int..

[12] Li L., Feng X., Teng F., Geng M., Li Y. (2025). Insights into Beeswax-Gelatin/Carboxymethyl Chitosan Bigel Systems: Structural-Property Relationships Governing Dual Encapsulation of Hydrophilic and Hydrophobic Bioactive Compounds. Food Chem..

[13] Keramat M., Ehsandoost E., Golmakani M.-T. (2023). Recent Trends in Improving the Oxidative Stability of Oil-Based Food Products by Inhibiting Oxidation at the Interfacial Region. Foods.

[14] Rasekhi Kazeruni A., Hosseini S.M.H., Keramat M., Niakousari M., Ghiasi F., Golmakani M.-T. (2025). Antioxidant Mechanism of Ferulic Acid and γ-Oryzanol in Emulsion and Emulgel Based on Black Seed Oil. Food Chem. X.

[15] Chen X.-W., Hu Q.-H., Li X.-X., Ma C.-G. (2022). Systematic Comparison of Structural and Lipid Oxidation in Oil-in-Water and Water-in-Oil Biphasic Emulgels: Effect of Emulsion Type, Oil-Phase Composition, and Oil Fraction. J. Sci. Food Agric..

[16] Hennebelle M., Villeneuve P., Durand E., Lecomte J., van Duynhoven J., Meynier A., Yesiltas B., Jacobsen C., Berton-Carabin C. (2024). Lipid Oxidation in Emulsions: New Insights from the Past Two Decades. Prog. Lipid Res..

[17] Azmoon E., Saberi F., Kouhsari F., Akbari M., Kieliszek M., Vakilinezam A. (2021). The Effects of Hydrocolloids-Protein Mixture as a Fat Replacer on Physicochemical Characteristics of Sugar-Free Muffin Cake: Modeling and Optimization. Foods.

[18] Bao Y., Pignitter M. (2023). Mechanisms of Lipid Oxidation in Water-in-oil Emulsions and Oxidomics-guided Discovery of Targeted Protective Approaches. Compr. Rev. Food Sci. Food Saf..

[19] Villeneuve P., Bourlieu-Lacanal C., Durand E., Lecomte J., McClements D.J., Decker E.A. (2023). Lipid Oxidation in Emulsions and Bulk Oils: A Review of the Importance of Micelles. Crit. Rev. Food Sci. Nutr..

[20] Hashemi B., Assadpour E., Wang Y., Jafari S.M. (2025). Application of Oleogels, Hydrogels and Bigels as Novel Edible Inks for 3D/4D Printing of Food Products. Adv. Colloid Interface Sci..

[21] Flöter E., Wettlaufer T., Conty V., Scharfe M. (2021). Oleogels—Their Applicability and Methods of Characterization. Molecules.

[22] Hou Y., Wu Y., Ouyang J. (2024). Novel Bigel Based on Nanocellulose Hydrogel and Monoglyceride Oleogel: Preparation, Characteristics and Application as Fat Substitute. Food Res. Int..

[23] Sarraf M., Naji-Tabasi S., Beig-Babaei A., Moros J.E., Carrillo M.C.S., Tenorio-Alfonso A. (2024). Developing Edible Oleogels Structure Prepared with Emulsion-Template Approach Based on Soluble Biopolymer Complex. Food Chem. X.

[24] Miao W., Zhang Z., Lin Q., McClements D.J., Ji H., Jiang L., Wen J., Jin Z., Qiu C. (2025). Preparation of Emulsion-Template Oleogels: Tuning Properties by Controlling Initial Water Content and Evaporation Method. Food Hydrocoll..

[25] Costa M., Losada-Barreiro S., Bravo-Díaz C., Vicente A.A., Monteiro L.S., Paiva-Martins F. (2020). Influence of AO Chain Length, Droplet Size and Oil to Water Ratio on the Distribution and on the Activity of Gallates in Fish Oil-in-Water Emulsified Systems: Emulsion and Nanoemulsion Comparison. Food Chem..

[26] Choe E., Lee J., Min D.B. (2005). Chemistry for Oxidative Stability of Edible Oils. Healthful Lipids.

[27] Nuchi C.D., McClements D.J., Decker E.A. (2001). Impact of Tween 20 Hydroperoxides and Iron on the Oxidation of Methyl Linoleate and Salmon Oil Dispersions. J. Agric. Food Chem..

[28] Silvestre M.P.C., Chaiyasit W., Brannan R.G., McClements D.J., Decker E.A. (2000). Ability of Surfactant Headgroup Size to Alter Lipid and Antioxidant Oxidation in Oil-in-Water Emulsions. J. Agric. Food Chem..

[29] Chaiyasit W., McClements D.J., Decker E.A. (2005). The Relationship between the Physicochemical Properties of Antioxidants and Their Ability to Inhibit Lipid Oxidation in Bulk Oil and Oil-in-Water Emulsions. J. Agric. Food Chem..

[30] Sato A.C.K., Moraes K.E.F.P., Cunha R.L. (2014). Development of Gelled Emulsions with Improved Oxidative and pH Stability. Food Hydrocoll..

[31] Gulcin İ., Alwasel S.H. (2022). Metal Ions, Metal Chelators and Metal Chelating Assay as Antioxidant Method. Processes.

[32] Branco G.F., Rodrigues M.I., Gioielli L.A., Castro I.A. (2011). Effect of the Simultaneous Interaction among Ascorbic Acid, Iron and pH on the Oxidative Stability of Oil-in-Water Emulsions. J. Agric. Food Chem..

[33] El-Lateef H.M.A., El-Dabea T., Khalaf M.M., Abu-Dief A.M. (2023). Recent Overview of Potent Antioxidant Activity of Coordination Compounds. Antioxidants.

[34] García-Moreno P.J., Guadix A., Guadix E.M., Jacobsen C. (2016). Physical and Oxidative Stability of Fish Oil-in-Water Emulsions Stabilized with Fish Protein Hydrolysates. Food Chem..

[35] Ogawa S., Decker E.A., McClements D.J. (2003). Influence of Environmental Conditions on the Stability of Oil in Water Emulsions Containing Droplets Stabilized by Lecithin−Chitosan Membranes. J. Agric. Food Chem..

[36] Yuji H., Weiss J., Villeneuve P., López Giraldo L.J., Figueroa-Espinoza M.-C., Decker E.A. (2007). Ability of Surface-Active Antioxidants to Inhibit Lipid Oxidation in Oil-in-Water Emulsion. J. Agric. Food Chem..

[37] Saha S.K., Lee S.B., Won J., Choi H.Y., Kim K., Yang G.-M., Dayem A.A., Cho S. (2017). Correlation between Oxidative Stress, Nutrition, and Cancer Initiation. Int. J. Mol. Sci..

[38] Lakey-Beitia J., Kumar D.J., Hegde M.L., Rao K.S. (2019). Carotenoids as Novel Therapeutic Molecules Against Neurodegenerative Disorders: Chemistry and Molecular Docking Analysis. Int. J. Mol. Sci..

[39] Paroń O., Harasym J. (2025). Single-Gelator Structuring of Hemp Oil Using Agarose: Comparative Assembly, Electronic Nose Profiling, and Functional Performance of Hydroleogels Versus Oleogels in Shortbread Cookies. Polymers.

[40] Amft J., Meissner P.M., Steffen-Heins A., Hasler M., Stöckmann H., Meynier A., Birault L., Velasco J., Vermoesen A., Perez-Portabella I. (2023). Interlaboratory Study on Lipid Oxidation during Accelerated Storage Trials with Rapeseed and Sunflower Oil Analyzed by Conjugated Dienes as Primary Oxidation Products. Eur. J. Lipid Sci. Technol..

[41] Dridi W., Essafi W., Gargouri M., Leal-Calderon F., Cansell M. (2016). Influence of Formulation on the Oxidative Stability of Water-in-Oil Emulsions. Food Chem..

[42] Ropciuc S., Codina G.G., Oroian M.A., Dranca F., Leahu A., Prisacaru A.E. (2023). Formulation of Oleogels Based on Candelilla Wax: Physicochemical and Rheological Characterization. Proceedings of the 23rd International Multidisciplinary Scientific GeoConference SGEM, Vienna, Austria, 15 December 2023.

[43] Sun Y., Xiao C., Hu X., Ren J., Song C., Zhao Y. (2026). Influence of Composite Gelator-Based Oleogels as Fat Substitutes on Gel-Type Prepared Meatballs as a Meat Model System for Changes in Gel Formation, Physical Properties and Oxidation Stability. LWT.

[44] Pang M., Shi Z., Lei Z., Ge Y., Jiang S., Cao L. (2020). Structure and Thermal Properties of Beeswax-Based Oleogels with Different Types of Vegetable Oil. Grasas Aceites.

[45] Baltuonytė G., Eisinaitė V., Kazernavičiūtė R., Vinauskienė R., Jasutienė I., Leskauskaitė D. (2022). Novel Formulation of Bigel-Based Vegetable Oil Spreads Enriched with Lingonberry Pomace. Foods.

[46] Kmiecik D., Fedko M., Siger A., Kulczyński B. (2019). Degradation of Tocopherol Molecules and Its Impact on the Polymerization of Triacylglycerols during Heat Treatment of Oil. Molecules.

[47] Mora R.L., Vanare S.P., Pegg R.B. (2025). Mechanisms, Causes, and Solutions: A Comprehensive Review of Lipid Oxidation in Low-Moisture Packaged Snacks. Eur. J. Lipid Sci. Technol..

[48] Schaich K.M. (2024). Epoxides: An Underestimated Lipid Oxidation Product. Free Radic. Res..

[49] Mancebo-Campos V., Salvador M.D., Fregapane G. (2014). Antioxidant Capacity of Individual and Combined Virgin Olive Oil Minor Compounds Evaluated at Mild Temperature (25 and 40 °C) as Compared to Accelerated and Antiradical Assays. Food Chem..

[50] Grootveld M., Percival B.C., Leenders J., Wilson P.B. (2020). Potential Adverse Public Health Effects Afforded by the Ingestion of Dietary Lipid Oxidation Product Toxins: Significance of Fried Food Sources. Nutrients.

[51] Shaikh S., Nazam N., Rizvi S.M.D., Ahmad K., Baig M.H., Lee E.J., Choi I. (2019). Mechanistic Insights into the Antimicrobial Actions of Metallic Nanoparticles and Their Implications for Multidrug Resistance. Int. J. Mol. Sci..

[52] Rehman A., Ahmad T., Aadil R.M., Spotti M.J., Bakry A.M., Khan I.M., Zhao L., Riaz T., Tong Q. (2019). Pectin Polymers as Wall Materials for the Nano-Encapsulation of Bioactive Compounds. Trends Food Sci. Technol..

[53] Huang Y., Li C., McClements D.J. (2025). Recent Advances in Plant-Based Emulsion Gels: Preparation, Characterization, Applications, and Future Perspectives. Gels.

[54] Grebenteuch S., Kroh L.W., Drusch S., Rohn S. (2021). Formation of Secondary and Tertiary Volatile Compounds Resulting from the Lipid Oxidation of Rapeseed Oil. Foods.

[55] Pignitter M., Somoza V. (2020). Critical Evaluation of Methods for the Measurement of Oxidative Rancidity in Vegetable Oils. J. Food Drug Anal..

[56] Zhao M., Liu Z., Zhang W., Xia G., Li C., Rakariyatham K., Zhou D. (2025). Advance in Aldehydes Derived from Lipid Oxidation: A Review of the Formation Mechanism, Attributable Food Thermal Processing Technology, Analytical Method and Toxicological Effect. Food Res. Int..

[57] Akshaya C., Sindhuja V., Aarthi D., Suganya K., Sathish A. (2026). Bigels as a Platform for Advanced Topical Application. Int. J. Pharm. Res. Dev..

[58] Shakouri S., Tehrani M.M., Koocheki A., Farhoosh R., Abdolshahi A., Shariatifar N. (2025). Bigels as Novel Drug Delivery Systems: A Systematic Review on Efficiency and Influential Factors. Curr. Rev. Clin. Exp. Pharmacol..

[59] Singh V.K., Banerjee I., Agarwal T., Pramanik K., Bhattacharya M.K., Pal K. (2014). Guar Gum and Sesame Oil Based Novel Bigels for Controlled Drug Delivery. Colloids Surf. B Biointerfaces.

[60] Moreno-Caballero M., Ortega-Barbosa J.P., Palomeque-Forero L.A., Lizarazo-Aparicio M.C., Miranda-Lasprilla D., Ballesteros-Vivas D., Parada-Alfonso F., Ibañez-Ezequiel E. (2026). Development and Characterization of Oleogels from Avocado Oil and Monoglycerides. Foods.

[61] Hashemi B., Assadpour, Jafari S.M. (2026). Oleogels for Development of Future Food Products Based on Health Orientation: Research Progress, Challenges and Applications. Food Hydrocoll. Health.

[62] Dimakopoulou-Papazoglou D., Zampouni K., Prodromidis P., Moschakis T., Katsanidis E. (2024). Microstructure, Physical Properties, and Oxidative Stability of Olive Oil Oleogels Composed of Sunflower Wax and Monoglycerides. Gels.

[63] Pullar J.M., Carr A.C., Vissers M.C.M. (2017). The Roles of Vitamin C in Skin Health. Nutrients.

[64] Cengiz A., Kahyaoglu T., Schröen K., Berton-Carabin C. (2019). Oxidative Stability of Emulsions Fortified with Iron: The Role of Liposomal Phospholipids. J. Sci. Food Agric..

[65] Jacobsen C., Hartvigsen K., Lund P., Meyer A.S., Adler-Nissen J., Holstborg J., Hølmer G. (1999). Oxidation in Fish-Oil-Enriched Mayonnaise1. Assessment of Propyl Gallate as an Antioxidant by Discriminant Partial Least Squares Regression Analysis. Eur. Food Res. Technol..

[66] Hermund D.B., Yeşiltaş B., Honold P., Jónsdóttir R., Kristinsson H.G., Jacobsen C. (2015). Characterisation and Antioxidant Evaluation of Icelandic *F. vesiculosus* Extracts in Vitro and in Fish-Oil-Enriched Milk and Mayonnaise. J. Funct. Foods.

[67] Let M.B., Jacobsen C., Meyer A.S. (2007). Lipid Oxidation in Milk, Yoghurt, and Salad Dressing Enriched with Neat Fish Oil or Pre-Emulsified Fish Oil. J. Agric. Food Chem..

[68] Shamsuddin N.A.M., Zulfakar M.H. (2023). Nanostructured Lipid Carriers for the Delivery of Natural Bioactive Compounds. Curr. Drug Deliv..

[69] Ghiasi A., Zampouni K., Mourtzinos I., Katsanidis E. (2022). Hydrogels, Oleogels and Bigels as Edible Coatings of Sardine Fillets and Delivery Systems of Rosemary Extract. Gels.

[70] Yu H., Huang G., Ma Y., Liu Y., Huang X., Zheng Q., Yue P., Yang M. (2021). Cellulose Nanocrystals Based Clove Oil Pickering Emulsion for Enhanced Antibacterial Activity. Int. J. Biol. Macromol..

[71] Lukešová D., Dostálová J., El-Moneim Mahmoud E., Svárovská M. (2009). Oxidation Changes of Vegetable Oils during Microwave Heating. Czech J. Food Sci..

[72] Yin B.-X., Zhang Y.-F., Hou X.-Y., Zhao X.-Y., Luo Y.-Q., Cai X.-S., Liu H.-M., Zhu X.-L., Wang X.-D. (2025). Influence of Unsaturated Fatty Acids on the Microstructural and Functional Properties of Sunflower Wax-Based Oleogel. LWT.

[73] Khalid N., Kobayashi I., Neves M.A., Uemura K., Nakajima M., Nabetani H. (2015). Monodisperse Aqueous Microspheres Encapsulating High Concentration of L-Ascorbic Acid: Insights of Preparation and Stability Evaluation from Straight-through Microchannel Emulsification. Biosci. Biotechnol. Biochem..

[74] Ramadhan A.H., Yu D., Hlaing K.S.S., Jiang Q., Xu Y., Xia W. (2025). Effects of Packaging and Storage Time on Lipid and Protein Oxidation and Modifications in Texture Characteristics of Refrigerated Grass Carp (*Ctenopharyngodon idellus*) Fish Muscles. Int. J. Food Sci. Technol..

[75] Nishida Y., Berg P.C., Shakersain B., Hecht K., Takikawa A., Tao R., Kakuta Y., Uragami C., Hashimoto H., Misawa N. (2023). Astaxanthin: Past, Present, and Future. Mar. Drugs.

[76] Shahidi F., Ambigaipalan P. (2015). Phenolics and Polyphenolics in Foods, Beverages and Spices: Antioxidant Activity and Health Effects—A Review. J. Funct. Foods.

[77] Abou-Elsoud M., Khalifa I., Salama M., Li Z., Zahran H., Cai Z., Ahn D.U., Huang X. (2026). Impact of Fatty Acid Unsaturation Levels on the Physicochemical Properties and Oxidative/Digestive Stability of Pectin/LDL-Based Oleogels. Food Hydrocoll..

[78] Guo J., Cui L., Meng Z. (2023). Oleogels/Emulsion Gels as Novel Saturated Fat Replacers in Meat Products: A Review. Food Hydrocoll..

[79] Li L., Liu G., Bogojevic O., Pedersen J.N., Guo Z. (2022). Edible Oleogels as Solid Fat Alternatives: Composition and Oleogelation Mechanism Implications. Comp. Rev. Food Sci. Food Saf..

[80] Harasym J., Paroń O., Pejcz E. (2025). Pea Protein Isolates: From Extraction to Functionality. Molecules.

[81] Shantha N.C., Decker E.A. (1994). Rapid, Sensitive, Iron-Based Spectrophotometric Methods for Determination of Peroxide Values of Food Lipids. J. AOAC Int..

